# Rare case of gastric inflammatory fibroid polyp located at the fornix of the stomach and mimicking gastric cancer: a case report

**DOI:** 10.1186/s40792-020-00980-0

**Published:** 2020-11-23

**Authors:** Akimasa Kawai, Hideo Matsumoto, Ken Haruma, Tomoko Kanzaki, Yuji Sugawara, Takashi Akiyama, Toshihiro Hirai

**Affiliations:** 1 Department of Surgery, Mitsugi General Hospital, 124, Ichi, Mitsugi-cho, Onomichi, Hiroshima 722-0393 Japan; 2 Department of General Internal Medicine 2, Kawasaki Medical School General Medical Center, 2-6-1, Nakasange, Kita-ku, Okayama, Okayama 700-8505 Japan; 3 Department of Internal Medicine, Mitsugi General Hospital, 124, Ichi, Mitsugi-cho, Onomichi, Hiroshima 722-0393 Japan; 4 Department of Pathology, Kawasaki Medical School, 577, Matsushima, Kurasiki, Okayama 701-0192 Japan

**Keywords:** Inflammatory fibroid polyp, An ulcerated tumor, The fornix of stomach

## Abstract

**Background:**

Gastric inflammatory fibroid polyp (IFP) is a rare polypoid lesion of the stomach that is characterized pathologically by the presence of spindle cells, a prominent network of blood vessels, and inflammatory infiltration of eosinophils. IFP is mainly located in the gastric antrum and is usually semi-pedunculated and covered with normal mucosa. There have been several reports of large IFPs with ulceration on the surface, at the apex, but no report of the IFP with ulceration at the fornix of the stomach. We report a case of IFP with ulceration that was suggested to be gastric cancer and was resected for diagnostic treatment.

**Case presentation:**

A 79-year-old woman presented to our hospital. During mass screening for cancer, stomach fluoroscopy revealed an abnormal shadow. Endoscopy showed an ulcerated tumor at the fornix of stomach; hence, gastric cancer was suggested because of the polypoid lesion with irregular ridges and ulceration. Pathological diagnosis of gastric biopsy specimens revealed an inflammation of the gastric mucosa, and specific findings for gastric cancer were not obtained. Because we could not exclude gastric malignancies such as cancer or gastrointestinal stromal tumor, we performed a partial resection of the stomach with a 2-cm margin using the laparoscopic-assisted method. Pathological examination of the resected specimen revealed that the tumor was present in the submucosal layer and consisted of collagen fiber containing inflammatory cell infiltration of mainly eosinophils. A prominent network of blood vessels was also found in the specimens. Immunohistochemical staining revealed mild positivity for CD34, and α-SMA and was negative for c-kit, DOG-1, s-100, desmin, ALK, and IgG4. The lesion was thus diagnosed as an IFP. The postoperative course was uneventful. The patient is currently asymptomatic and has shown no recurrence.

**Conclusion:**

IFPs have variable locational, morphological, histological, pathological, and immunohistochemical features. We reported that the gastric IFP was located at the fornix of the stomach and was similar in morphology to gastric cancer. This case is clinically significant to avoid over-surgery.

## Background

Inflammatory fibroid polyps (IFPs) are benign polypoid lesions of the stomach. They are typically noninvasive, non-ulcerated, and eosinophilic submucosal tumors (SMT) with spindle cell proliferation. They occur in a variety of sites in the gastrointestinal tract, but the most common site of IFP is the gastric antrum [1–5]. In 143 cases of IFP, Stolte reported that the polyp was located at the antrum in 77.6%, in the angular notch region in 9.8%, in the pylorus in 1.4%, and in the fundus and cardia in only 0.7% [1]. It has been reported that the IFP tends to form ulcers at the apex as it grows larger. Here, we report a rare case of gastric IFP that was located at the fornix and formed a central ulceration thereby mimicking gastric cancer.

## Case presentation

**Fig. 1:**
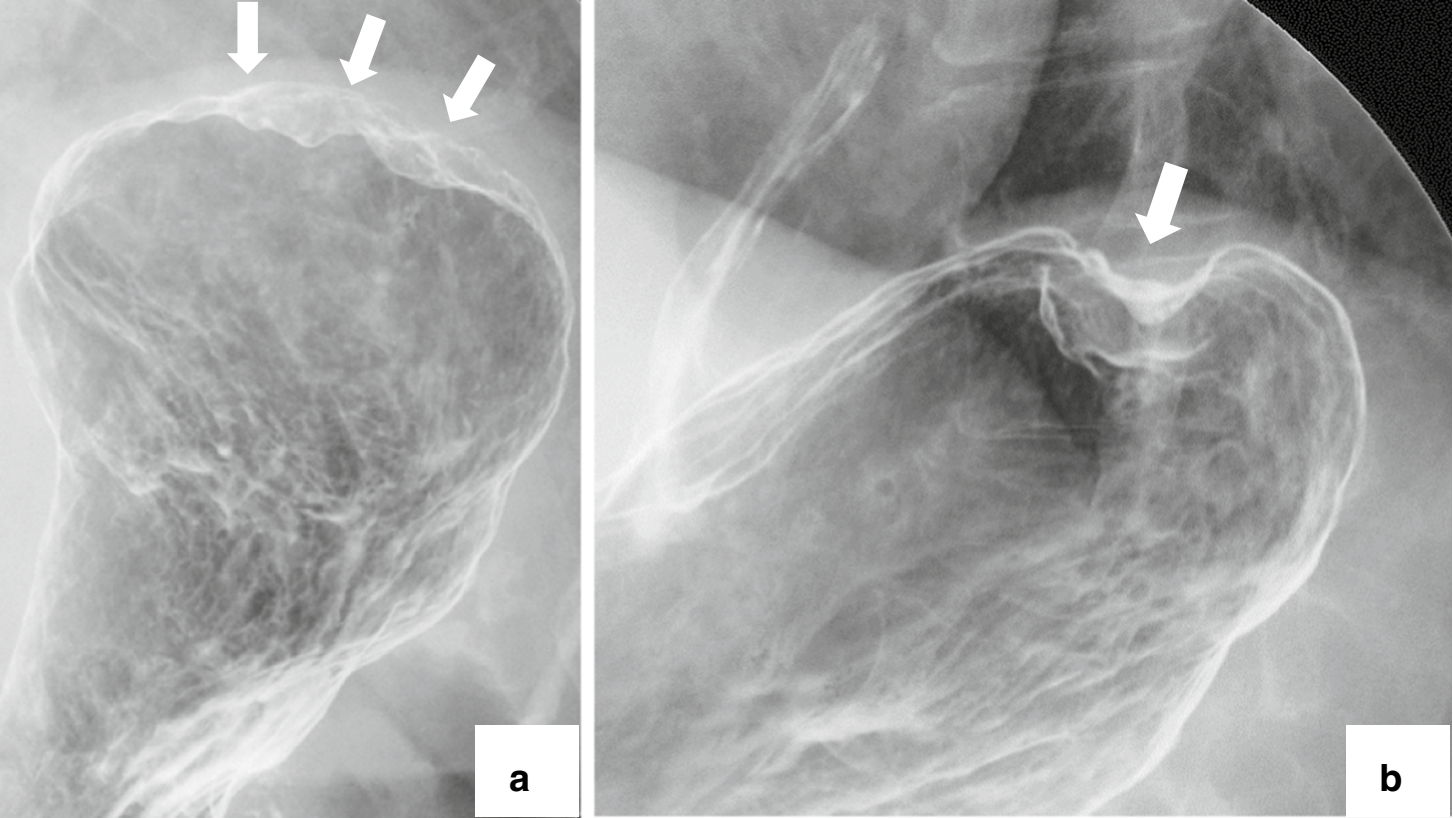
Stomach fluoroscopic findings at mass screening. Irregular filling defects found at the fornix of the stomach (**a**; arrows). The second oblique position shows a polypoid lesion with an irregular margin (**b**; arrow)

**Fig. 2:**
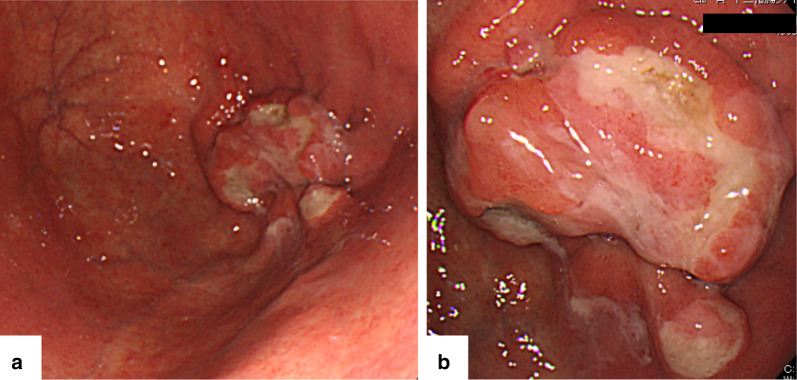
Endoscopy reveals an ulcerated tumor at the fornix of the stomach (**a**). Close-up finding indicates a polypoid lesion with an irregular ulcer with white lichen (**b**)

**Fig. 3:**
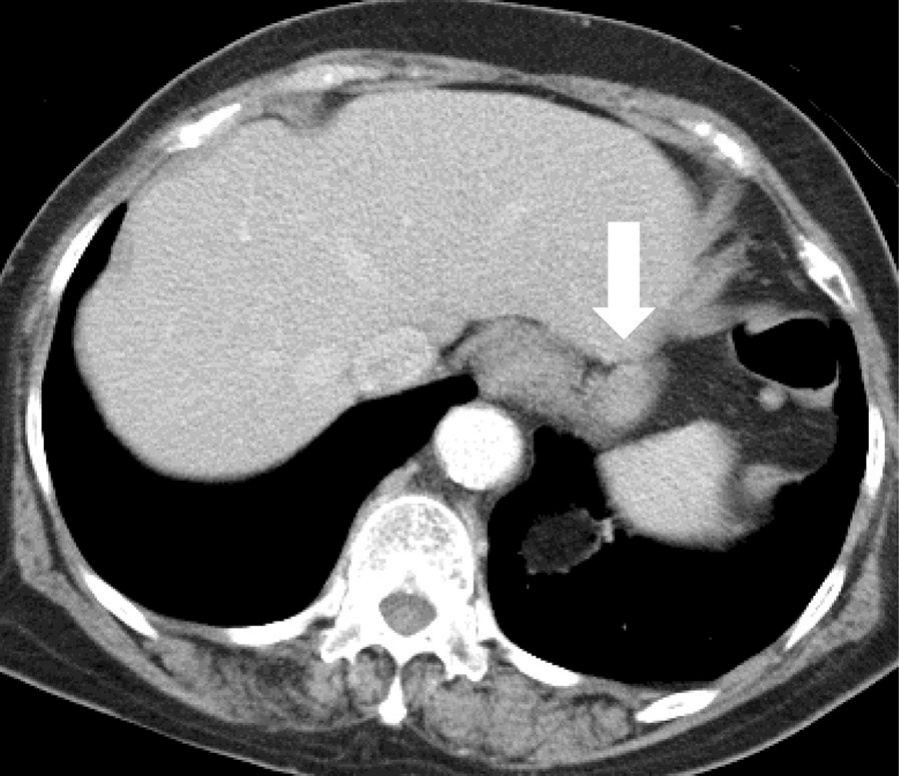
Computed tomography scan reveals an iso-enhancing and well-defined nodule (approximately 16 × 13 mm) protruding from the upper edge at the fornix (arrow)

**Fig. 4:**
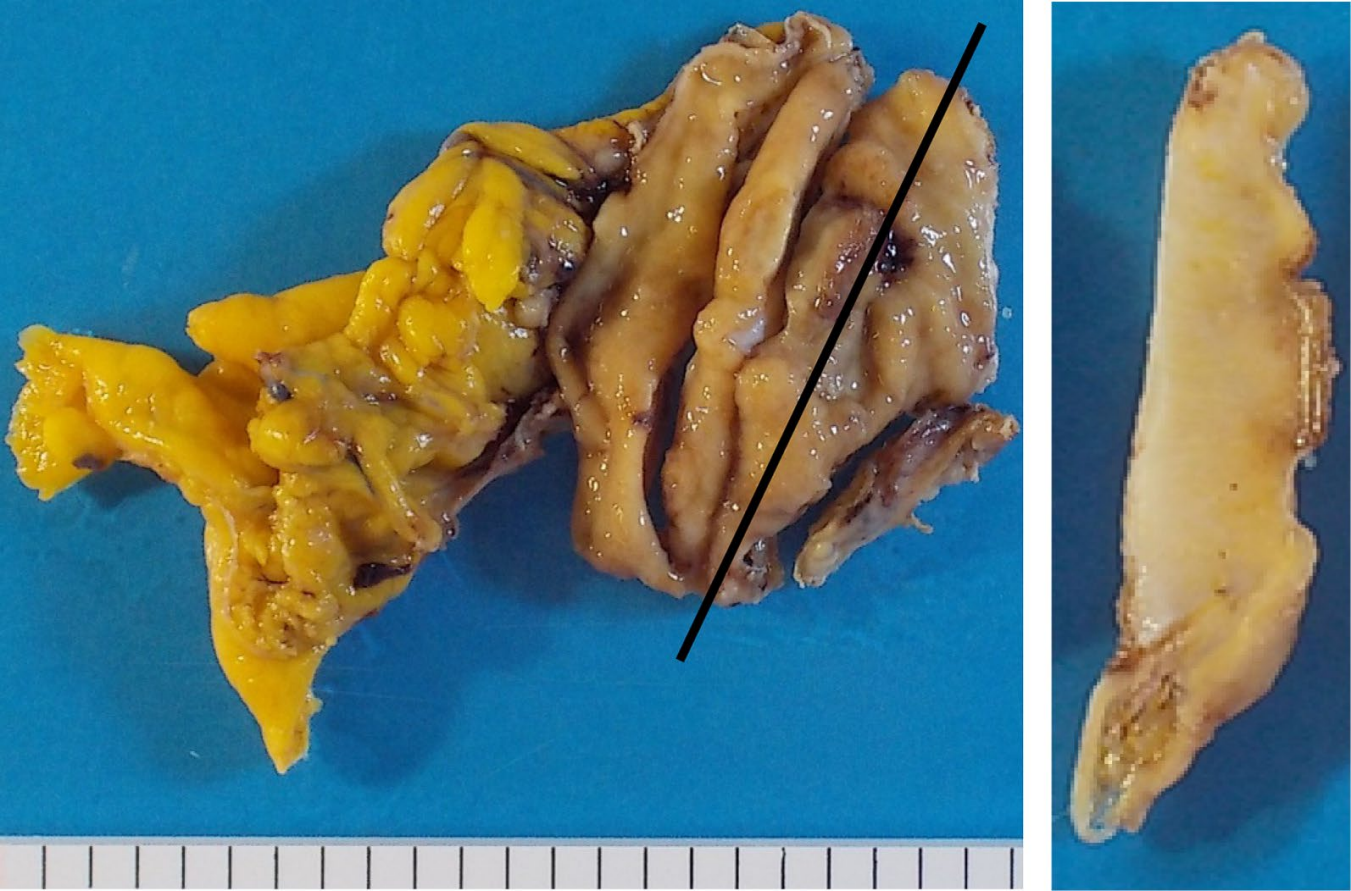
Resected specimen reveals central ulceration of the polypoid lesion (about 30 × 30 mm). Black line is cut line. The cut part reveals a solid tumor covered with normal mucosa

**Fig. 5:**
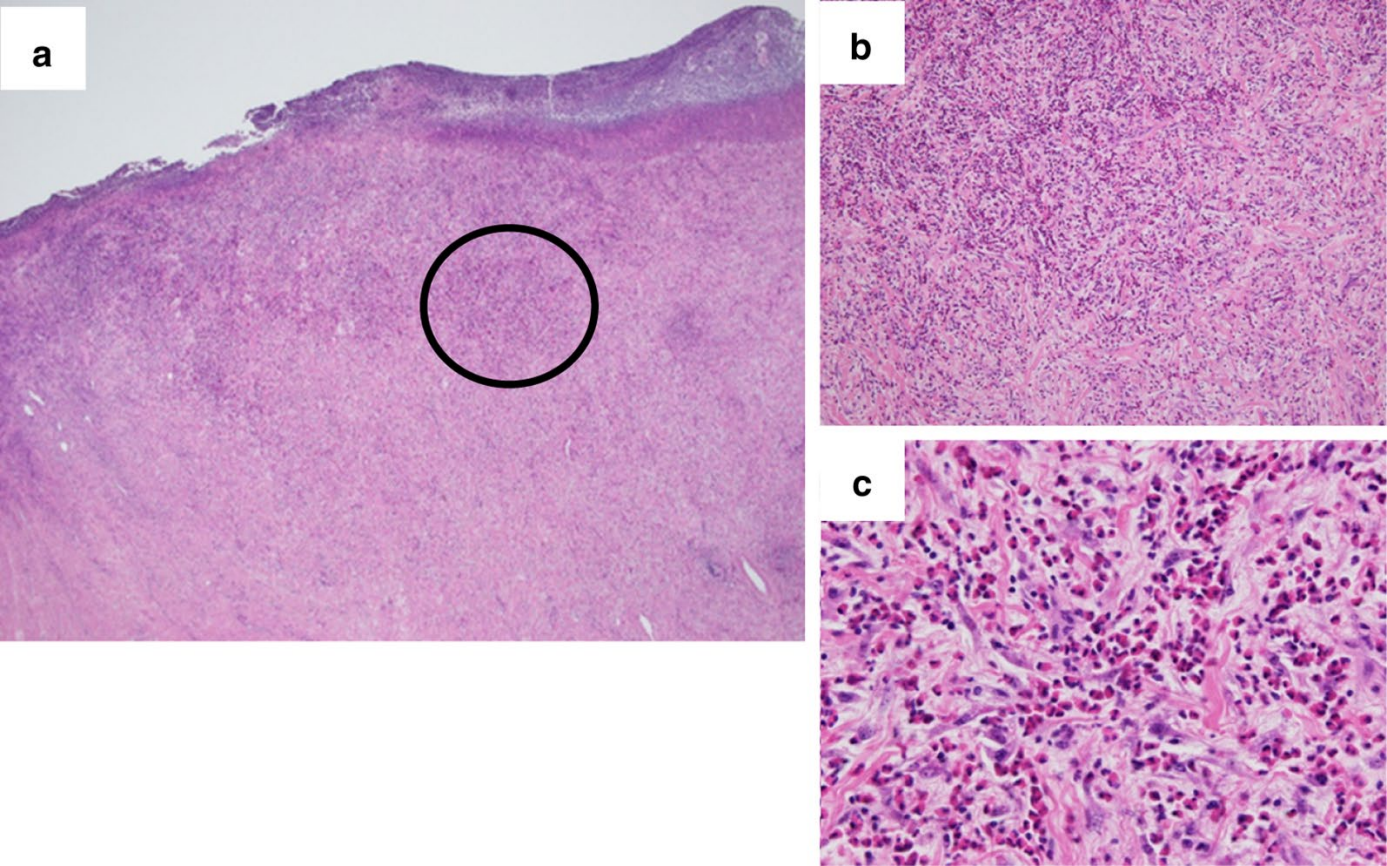
Pathological examination reveals that the tumor exists mainly in the submucosal layer without atypia of the mucosa (**a**). **b**, **c** Views expand the black circle. They consist of collagen fibers containing inflammatory cells, plasma cells, and eosinophilic cell infiltration (**b**, **c**)

A 79-year-old woman presented to our hospital. An abnormal shadow using stomach fluoroscopy was detected during mass screening for gastric cancer. The examination showed an irregular-shaped defect with a central ulceration at the fornix of the stomach (Fig. 1). CEA, CA19-9, and IL-2R were 1.7 ng/ml (normal range 0 to 6), 21.5 ng/ml (normal range 0 to 37), and 472 U/ml (normal range 127 to 582). Endoscopy revealed a polypoid lesion comprising irregular ridges with ulceration at the fornix of the stomach. The lesion was suggested to be a gastric malignancy such as gastric cancer, lymphoma, or gastrointestinal stromal tumor (GIST) (Fig. 2). A computed tomography scan revealed a nodule (approximately 16 × 13 mm) protruding from the upper edge of the fornix without lymphoid swelling and distant metastasis (Fig. 3). Family history of IFP was not found in this case. Pathological diagnosis of biopsy specimens revealed an ulceration with inflammatory cells in the necrotic tissue and granulation tissue. As we could not exclude a gastric malignancy, especially gastric cancer, we performed a partial resection of the stomach using the laparoscopic-assisted method. That surgery was performed with five ports (12 mm ports at the umbilicus and right-side abdomen, 5 mm ports at the bilateral upper abdomen and left-side abdomen). We cut off the greater omentum, left gastroepiploic artery/vein and posterior gastric artery/vein by the energy device. We transferred from the laparoscopic operation to open surgery. We confirmed the main lesion at the fornix of the stomach and performed a partial resection of the stomach with a 2-cm margin. The resected specimens revealed a polypoid lesion with indistinct margins and central ulceration (30 × 30 mm) (Fig. 4). Pathological examination revealed that the lesion existed in the submucosal layer without atypia of mucosa and consisted of collagen fibers containing inflammatory cells, plasma cells, and eosinophilic cell infiltration (Fig. 5a). Immunohistochemical staining was mildly positive for CD34 and α-SMA and negative for c-kit, DOG-1, s-100, desmin, ALK, and IgG4; the lesion was thus diagnosed as an IFP (Fig. 5b). No malignant lesion was not found in the surgical specimens. The postoperative course was uneventful. The patient is currently asymptomatic and has shown no recurrence.

## Conclusions and discussion

IFP is a rare benign polypoid lesion of the gastrointestinal tract. In the very first report in 1937, Kaijser described the gastric lesion with eosinophilic cell infiltration [6]. The lesions were described as gastric submucosal granuloma with eosinophilic infiltration by Vanek in 1949 [7]. Helwig et al. established the term IFP in 1953 [8].

The most common localization of IFP is the antrum of the stomach [1–5], but a few cases occur in the small bowel, large bowel, duodenum, appendix, and esophagus [5]. Typical endoscopic findings are shown as intraluminal, protruding, solitary polypoid or sessile, intramural lesion, with a smooth and often ulcerated mucosa, located in the antrum [8]. This case was located at the fornix of the stomach and endoscopy revealed an irregular polypoid lesion with ulceration at the fornix of the stomach, and the lesion was suggested to be a gastric malignancy such as gastric cancer, lymphoma, or GIST. The pathological diagnosis using gastric biopsy specimens revealed it to be benign. As we could not exclude gastric malignancy and considered complications such as easy hemorrhage, we performed a partial resection of the stomach with a 2-cm margin using the laparoscopic-assisted method.

Pathologically, IFP is characterized as a submucosal lesion containing spindle cells with a prominent vasculature and inflammatory cell infiltration, mainly eosinophilic cells. A specific feature is a perivascular “onion skin” appearance, but approximately half of IFPs do not have this appearance [9]. Our present case did not have an “onion skin” appearance.

**Fig. 6:**
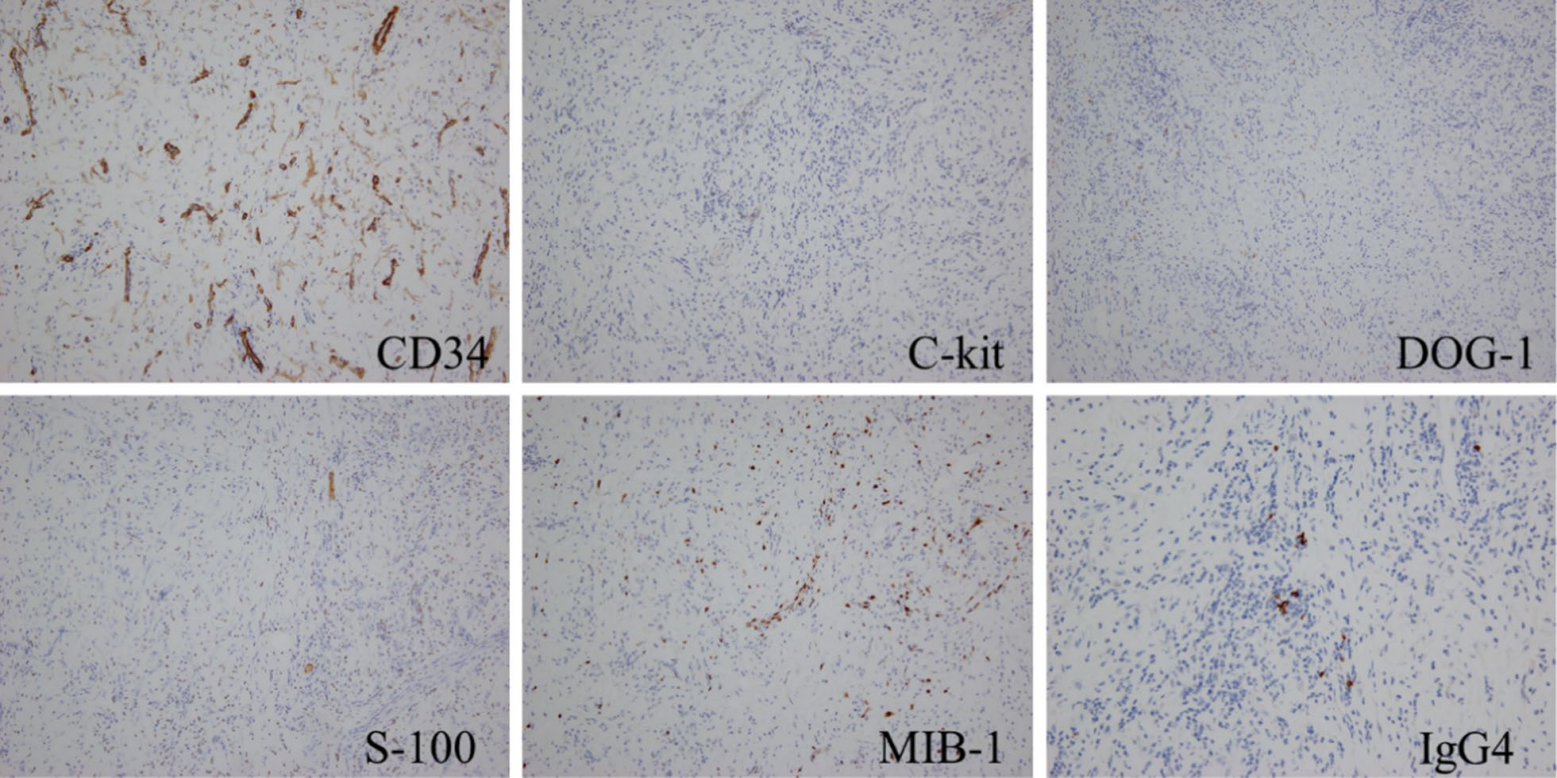
Immunohistochemical staining mildly positive for CD34 and negative for c-kit, DOG-1, s-100, MIB-1, and IgG4

Immunohistochemically, IFP was positive for CD34 and negative for c-kit, DOG-1, s-100, and desmin, and ALK. In the present case, the α-SMA was mildly positive only in fibrobrast cells and negative in spindle tumor cells. Recently IgG4 related lesions of the stomach have attracted attention for the ulcerative lesions of the stomach, which are difficult to diagnose; IgG4 contained cells were not found histochemically (Fig. 6).

Although several treatment options except the operation including re-biopsy, endoscopic resection, and follow-up were present, we selected the surgical operation to prevent bleeding from the tumor and to rule out the malignancies. Endoscopic resection was judged to an be-off label lesion from the shape and size of tumor. In general, the treatment of IFP depends on the shape, size, and location of the lesion. The current standard treatment is complete resection by endoscopy or gastric surgery, because it is considered as benign and noninvasive lesions [10–12]. However, a few cases have been observed to invade the muscularis propria layer and may exhibit local recurrence after inadequate resection [10]. In the present case, the spindle tumor cells did not invade the muscularis propria layer, and we resected the lesion with adequate margins.

Etiology and pathogenesis of IFP remain unclear. The local inflammatory response underlying an underlying granuloma such as trauma, tuberculosis, Crohn’s disease, and sarcoidosis were reported. In our case, the history of trauma, tuberculosis, Crohn’s disease and sarcoidosis was not recognized. Because *Helicobacter pylori* (*H. pylori*) was detected on the surface of gastric epithelium by Giemsa staining using the surgical materials, the association with IFP and *H. pylori* infection was not excluded [13]. Familial aggregation was reported previously, but it was not noted in our case [14]. In 2008, Schildhaus et al. identified the platelet-derived growth factor receptor alpha (PDGFRA) mutations in IFPs and these mutations had previously been detected only in GISTs [15]. The presence of PDGFRA mutations provides strong evidence of clonal proliferation and suggests that IFPs have a neoplastic nature. In this case, we did not test the mutations of PDGFRA.

In conclusion, we presented a rare case of IFP with ulceration mimicking gastric malignancies at the fornix of the stomach, which was treated using laparoscopic-assisted partial resection.

## Data Availability

All data are contained within the manuscript file.

## Abbreviations

IFP: Gastric inflammatory fibroid polyp
GIST: Gastrointestinal stromal tumor
